# 2-Ethyl-5-triphenyl­methyl-1,3-dioxane

**DOI:** 10.1107/S1600536810036767

**Published:** 2010-09-18

**Authors:** Lin Yuan, Jiang-Hua Shi, Min Zhang, Seik Weng Ng

**Affiliations:** a Department of Biology and Chemistry, Hunan University of Science and Engineering, Yongzhou Hunan 425100, People’s Republic of China; b Department of Chemistry, University of Malaya, 50603 Kuala Lumpur, Malaysia

## Abstract

In the title compound, C_25_H_26_O_2_, the dioxane ring adopts a chair conformation with the two substituent groups occupying equatorial positions.

## Related literature

For the crystal structure of 2,2-dimethyl-5-triphenyl-1,3-dioxane, see: Zhang *et al.* (2009 ▶).
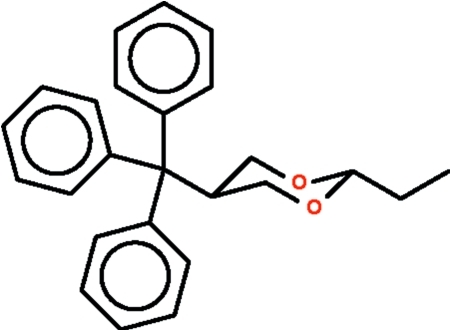


## Experimental

### 

#### Crystal data

C_25_H_26_O_2_
*M* *_r_* = 358.46
Monoclinic, 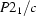

*a* = 10.5401 (6) Å
*b* = 13.3550 (8) Å
*c* = 14.6044 (8) Å
β = 110.523 (1)°
*V* = 1925.28 (19) Å^3^
*Z* = 4
Mo *K*α radiation
μ = 0.08 mm^−1^
*T* = 110 K
0.45 × 0.35 × 0.15 mm

#### Data collection

Bruker SMART APEX diffractometer
9493 measured reflections
4174 independent reflections
3047 reflections with *I* > 2σ(*I*)
*R*_int_ = 0.031

#### Refinement

*R*[*F* ^2^ > 2σ(*F* ^2^)] = 0.042
*wR*(*F* ^2^) = 0.114
*S* = 1.04
4174 reflections
244 parameters
H-atom parameters constrained
Δρ_max_ = 0.29 e Å^−3^
Δρ_min_ = −0.19 e Å^−3^

### 

Data collection: *SMART* (Bruker, 2003 ▶); cell refinement: *SAINT* (Bruker, 2003 ▶); data reduction: *SAINT*; program(s) used to solve structure: *SHELXS97* (Sheldrick, 2008 ▶); program(s) used to refine structure: *SHELXL97* (Sheldrick, 2008 ▶); molecular graphics: *X-SEED* (Barbour, 2001 ▶); software used to prepare material for publication: *publCIF* (Westrip, 2010 ▶).

## Supplementary Material

Crystal structure: contains datablocks global, I. DOI: 10.1107/S1600536810036767/zs2066sup1.cif

Structure factors: contains datablocks I. DOI: 10.1107/S1600536810036767/zs2066Isup2.hkl

Additional supplementary materials:  crystallographic information; 3D view; checkCIF report

## Figures and Tables

**Fig. 1.**
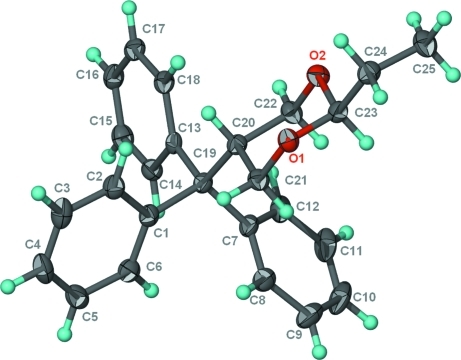
Thermal ellipsoid plot (Barbour, 2001) of the title compound at the 70% probability level.

## supplementary crystallographic information

### Comment

A previous study reported the crystal structure of 2,2-dimethyl-5-triphenylmethyl-1,3-dioxane (Zhang *et al.*, 2009). Such disubstituted 1,3-dioxanes are known from NMR studies to have substituents in equatorial rather than in axial orientations on the six-membered ring. The the title compound, 2-ethyl-5-triphenylmethyl-1,3-dioxane analog (Scheme I, Fig. 1), has similar features for the dioxane part, which adopts a chair conformation. The substitutent groups occupy equatorial positions.

### Experimental

2-Triphenylmethyl-1,3-propanediol (0.24 g, 5.0 mmol), propionaldehyde (20 mmol) and *p*-toluenesulfonic acid (0.1 g) were stirred in dichloromethane (20 ml) for a week. The solvent was evaporated and the residue was dissolved in ether (20 ml) after which the solution was washed with water and 5% sodium bicarbonate (20 ml). The organic phase was dried with anhydrous sodium sulfate. The solvent was evaporated and the product was recrystallized from ethyl acetate to give 1.0 g (yield 60%) of colorless crystals.

### Refinement

Carbon-bound H-atoms were placed in calculated positions (C—H = 0.95–0.99 Å) and were included in the refinement in the riding model approximation, with *U*_iso_(H) set to 1.2*U*_eq_(C).

### Crystal data

**Table:**

C_25_H_26_O_2_	*F*(000) = 768
*M**_r_* = 358.46	*D*_x_ = 1.237 Mg m^−^^3^
Monoclinic, *P*2_1_/*c*	Mo *K*α radiation, λ = 0.71073 Å
Hall symbol: -P 2ybc	Cell parameters from 3994 reflections
*a* = 10.5401 (6) Å	θ = 2.6–27.0°
*b* = 13.3550 (8) Å	µ = 0.08 mm^−^^1^
*c* = 14.6044 (8) Å	*T* = 110 K
β = 110.523 (1)°	Block, colorless
*V* = 1925.28 (19) Å^3^	0.45 × 0.35 × 0.15 mm
*Z* = 4	

### Data collection

**Table:**

Bruker SMART APEX diffractometer	3047 reflections with *I* > 2σ(*I*)
Radiation source: fine-focus sealed tube	*R*_int_ = 0.031
graphite	θ_max_ = 27.1°, θ_min_ = 2.1°
ω scans	*h* = −9→13
9493 measured reflections	*k* = −17→12
4174 independent reflections	*l* = −18→18

### Refinement

**Table:**

Refinement on *F*^2^	Primary atom site location: structure-invariant direct methods
Least-squares matrix: full	Secondary atom site location: difference Fourier map
*R*[*F*^2^ > 2σ(*F*^2^)] = 0.042	Hydrogen site location: inferred from neighbouring sites
*wR*(*F*^2^) = 0.114	H-atom parameters constrained
*S* = 1.04	*w* = 1/[σ^2^(*F*_o_^2^) + (0.0568*P*)^2^ + 0.2859*P*] where *P* = (*F*_o_^2^ + 2*F*_c_^2^)/3
4174 reflections	(Δ/σ)_max_ = 0.001
244 parameters	Δρ_max_ = 0.29 e Å^−^^3^
0 restraints	Δρ_min_ = −0.19 e Å^−^^3^

### Fractional atomic coordinates and isotropic or equivalent isotropic displacement parameters (Å^2^)

**Table:**

	*x*	*y*	*z*	*U*_iso_*/*U*_eq_	
O1	0.30810 (9)	0.32447 (7)	0.08575 (6)	0.0188 (2)	
O2	0.26796 (9)	0.32596 (7)	0.23220 (7)	0.0191 (2)	
C1	0.71880 (14)	0.43893 (10)	0.23149 (9)	0.0179 (3)	
C2	0.65864 (15)	0.52062 (11)	0.17152 (10)	0.0212 (3)	
H2	0.5682	0.5389	0.1633	0.025*	
C3	0.72914 (16)	0.57512 (11)	0.12403 (11)	0.0258 (3)	
H3	0.6864	0.6300	0.0835	0.031*	
C4	0.86148 (16)	0.55018 (12)	0.13528 (11)	0.0272 (4)	
H4	0.9093	0.5872	0.1022	0.033*	
C5	0.92283 (15)	0.47091 (12)	0.19517 (11)	0.0249 (3)	
H5	1.0137	0.4537	0.2038	0.030*	
C6	0.85276 (14)	0.41622 (11)	0.24288 (10)	0.0214 (3)	
H6	0.8968	0.3622	0.2841	0.026*	
C7	0.69636 (13)	0.27847 (10)	0.32337 (10)	0.0196 (3)	
C8	0.72962 (14)	0.21427 (11)	0.25990 (11)	0.0242 (3)	
H8	0.7284	0.2387	0.1985	0.029*	
C9	0.76470 (15)	0.11462 (12)	0.28526 (13)	0.0325 (4)	
H9	0.7870	0.0719	0.2411	0.039*	
C10	0.76718 (16)	0.07774 (12)	0.37422 (14)	0.0361 (4)	
H10	0.7914	0.0100	0.3915	0.043*	
C11	0.73423 (16)	0.13999 (12)	0.43758 (13)	0.0316 (4)	
H11	0.7355	0.1150	0.4988	0.038*	
C12	0.69899 (14)	0.23933 (11)	0.41254 (11)	0.0239 (3)	
H12	0.6763	0.2813	0.4570	0.029*	
C13	0.67391 (13)	0.45454 (10)	0.38294 (9)	0.0161 (3)	
C14	0.80413 (14)	0.45411 (11)	0.45377 (10)	0.0194 (3)	
H14	0.8701	0.4091	0.4470	0.023*	
C15	0.83889 (15)	0.51776 (11)	0.53341 (10)	0.0226 (3)	
H15	0.9278	0.5156	0.5807	0.027*	
C16	0.74504 (15)	0.58467 (11)	0.54476 (10)	0.0214 (3)	
H16	0.7689	0.6287	0.5993	0.026*	
C17	0.61592 (15)	0.58622 (11)	0.47519 (10)	0.0202 (3)	
H17	0.5504	0.6314	0.4824	0.024*	
C18	0.58085 (14)	0.52241 (10)	0.39474 (10)	0.0183 (3)	
H18	0.4921	0.5253	0.3473	0.022*	
C19	0.64436 (13)	0.38587 (10)	0.29220 (9)	0.0162 (3)	
C20	0.48869 (13)	0.37838 (10)	0.23326 (9)	0.0163 (3)	
H20	0.4528	0.4483	0.2205	0.020*	
C21	0.45229 (13)	0.32618 (11)	0.13438 (10)	0.0191 (3)	
H21A	0.4950	0.3620	0.0933	0.023*	
H21B	0.4876	0.2568	0.1440	0.023*	
C22	0.41008 (13)	0.32455 (11)	0.28894 (10)	0.0186 (3)	
H22A	0.4418	0.2545	0.3020	0.022*	
H22B	0.4263	0.3582	0.3525	0.022*	
C23	0.24357 (14)	0.27454 (11)	0.14269 (9)	0.0178 (3)	
H23A	0.2791	0.2046	0.1563	0.021*	
C24	0.09327 (14)	0.27184 (11)	0.08743 (10)	0.0207 (3)	
H24A	0.0758	0.2405	0.0227	0.025*	
H24B	0.0578	0.3412	0.0765	0.025*	
C25	0.01923 (15)	0.21322 (12)	0.14283 (11)	0.0261 (3)	
H25A	−0.0782	0.2131	0.1051	0.039*	
H25B	0.0354	0.2447	0.2066	0.039*	
H25C	0.0528	0.1442	0.1525	0.039*	

### Atomic displacement parameters (Å^2^)

**Table:**

	*U*^11^	*U*^22^	*U*^33^	*U*^12^	*U*^13^	*U*^23^
O1	0.0172 (5)	0.0204 (5)	0.0174 (5)	−0.0028 (4)	0.0042 (4)	−0.0008 (4)
O2	0.0159 (5)	0.0221 (5)	0.0181 (5)	−0.0013 (4)	0.0044 (4)	−0.0033 (4)
C1	0.0206 (7)	0.0169 (7)	0.0162 (6)	−0.0041 (6)	0.0063 (6)	−0.0047 (5)
C2	0.0219 (7)	0.0204 (7)	0.0207 (7)	−0.0027 (6)	0.0068 (6)	−0.0014 (6)
C3	0.0340 (9)	0.0213 (8)	0.0204 (7)	−0.0075 (7)	0.0076 (6)	0.0004 (6)
C4	0.0339 (9)	0.0294 (9)	0.0226 (7)	−0.0150 (7)	0.0152 (7)	−0.0060 (6)
C5	0.0219 (8)	0.0283 (8)	0.0267 (8)	−0.0083 (6)	0.0113 (6)	−0.0099 (6)
C6	0.0224 (7)	0.0198 (7)	0.0218 (7)	−0.0034 (6)	0.0076 (6)	−0.0056 (6)
C7	0.0137 (7)	0.0143 (7)	0.0275 (7)	−0.0004 (5)	0.0031 (6)	−0.0001 (6)
C8	0.0158 (7)	0.0200 (7)	0.0330 (8)	−0.0008 (6)	0.0039 (6)	−0.0045 (6)
C9	0.0188 (8)	0.0197 (8)	0.0509 (11)	0.0021 (6)	0.0019 (7)	−0.0099 (7)
C10	0.0217 (8)	0.0151 (8)	0.0569 (11)	0.0009 (6)	−0.0045 (8)	0.0038 (8)
C11	0.0225 (8)	0.0232 (8)	0.0393 (9)	−0.0026 (7)	−0.0016 (7)	0.0110 (7)
C12	0.0196 (7)	0.0192 (7)	0.0287 (8)	−0.0020 (6)	0.0034 (6)	0.0038 (6)
C13	0.0191 (7)	0.0122 (7)	0.0176 (6)	−0.0023 (5)	0.0072 (5)	0.0021 (5)
C14	0.0175 (7)	0.0193 (7)	0.0218 (7)	0.0001 (6)	0.0074 (6)	0.0014 (6)
C15	0.0197 (7)	0.0250 (8)	0.0208 (7)	−0.0045 (6)	0.0042 (6)	0.0001 (6)
C16	0.0278 (8)	0.0189 (7)	0.0187 (7)	−0.0053 (6)	0.0096 (6)	−0.0028 (6)
C17	0.0252 (8)	0.0160 (7)	0.0213 (7)	0.0012 (6)	0.0106 (6)	0.0028 (6)
C18	0.0190 (7)	0.0168 (7)	0.0182 (7)	−0.0001 (6)	0.0053 (5)	0.0024 (5)
C19	0.0160 (7)	0.0139 (7)	0.0178 (7)	0.0002 (5)	0.0047 (5)	0.0006 (5)
C20	0.0158 (7)	0.0152 (7)	0.0166 (7)	0.0005 (5)	0.0042 (5)	−0.0003 (5)
C21	0.0165 (7)	0.0203 (7)	0.0200 (7)	−0.0014 (6)	0.0059 (6)	−0.0020 (6)
C22	0.0151 (7)	0.0217 (7)	0.0175 (7)	−0.0013 (6)	0.0039 (5)	0.0006 (6)
C23	0.0200 (7)	0.0150 (7)	0.0185 (7)	−0.0011 (6)	0.0067 (6)	−0.0012 (5)
C24	0.0198 (7)	0.0196 (7)	0.0204 (7)	−0.0001 (6)	0.0042 (6)	−0.0022 (6)
C25	0.0193 (7)	0.0286 (8)	0.0286 (8)	−0.0044 (6)	0.0064 (6)	−0.0006 (7)

### Geometric parameters (Å, °)

**Table:**

O1—C23	1.4125 (16)	C13—C18	1.3899 (19)
O1—C21	1.4345 (15)	C13—C14	1.3994 (19)
O2—C23	1.4181 (15)	C13—C19	1.5509 (18)
O2—C22	1.4355 (15)	C14—C15	1.382 (2)
C1—C6	1.3959 (19)	C14—H14	0.9500
C1—C2	1.403 (2)	C15—C16	1.385 (2)
C1—C19	1.5465 (19)	C15—H15	0.9500
C2—C3	1.387 (2)	C16—C17	1.384 (2)
C2—H2	0.9500	C16—H16	0.9500
C3—C4	1.387 (2)	C17—C18	1.3925 (19)
C3—H3	0.9500	C17—H17	0.9500
C4—C5	1.381 (2)	C18—H18	0.9500
C4—H4	0.9500	C19—C20	1.5661 (18)
C5—C6	1.387 (2)	C20—C21	1.5264 (18)
C5—H5	0.9500	C20—C22	1.5287 (19)
C6—H6	0.9500	C20—H20	1.0000
C7—C8	1.395 (2)	C21—H21A	0.9900
C7—C12	1.395 (2)	C21—H21B	0.9900
C7—C19	1.5459 (19)	C22—H22A	0.9900
C8—C9	1.396 (2)	C22—H22B	0.9900
C8—H8	0.9500	C23—C24	1.5053 (18)
C9—C10	1.381 (3)	C23—H23A	1.0000
C9—H9	0.9500	C24—C25	1.523 (2)
C10—C11	1.376 (2)	C24—H24A	0.9900
C10—H10	0.9500	C24—H24B	0.9900
C11—C12	1.392 (2)	C25—H25A	0.9800
C11—H11	0.9500	C25—H25B	0.9800
C12—H12	0.9500	C25—H25C	0.9800
			
C23—O1—C21	111.18 (10)	C15—C16—H16	120.6
C23—O2—C22	109.94 (10)	C16—C17—C18	120.80 (13)
C6—C1—C2	117.39 (13)	C16—C17—H17	119.6
C6—C1—C19	121.83 (12)	C18—C17—H17	119.6
C2—C1—C19	120.33 (12)	C13—C18—C17	120.90 (13)
C3—C2—C1	121.06 (14)	C13—C18—H18	119.6
C3—C2—H2	119.5	C17—C18—H18	119.6
C1—C2—H2	119.5	C7—C19—C1	113.28 (11)
C4—C3—C2	120.50 (14)	C7—C19—C13	110.66 (11)
C4—C3—H3	119.7	C1—C19—C13	103.22 (10)
C2—C3—H3	119.7	C7—C19—C20	107.31 (11)
C5—C4—C3	119.17 (14)	C1—C19—C20	111.01 (10)
C5—C4—H4	120.4	C13—C19—C20	111.43 (11)
C3—C4—H4	120.4	C21—C20—C22	106.57 (11)
C4—C5—C6	120.53 (14)	C21—C20—C19	114.62 (11)
C4—C5—H5	119.7	C22—C20—C19	113.35 (10)
C6—C5—H5	119.7	C21—C20—H20	107.3
C5—C6—C1	121.34 (14)	C22—C20—H20	107.3
C5—C6—H6	119.3	C19—C20—H20	107.3
C1—C6—H6	119.3	O1—C21—C20	110.30 (11)
C8—C7—C12	117.57 (14)	O1—C21—H21A	109.6
C8—C7—C19	121.37 (13)	C20—C21—H21A	109.6
C12—C7—C19	120.78 (13)	O1—C21—H21B	109.6
C7—C8—C9	120.98 (15)	C20—C21—H21B	109.6
C7—C8—H8	119.5	H21A—C21—H21B	108.1
C9—C8—H8	119.5	O2—C22—C20	109.71 (10)
C10—C9—C8	120.36 (16)	O2—C22—H22A	109.7
C10—C9—H9	119.8	C20—C22—H22A	109.7
C8—C9—H9	119.8	O2—C22—H22B	109.7
C11—C10—C9	119.42 (15)	C20—C22—H22B	109.7
C11—C10—H10	120.3	H22A—C22—H22B	108.2
C9—C10—H10	120.3	O1—C23—O2	110.18 (10)
C10—C11—C12	120.42 (16)	O1—C23—C24	109.22 (11)
C10—C11—H11	119.8	O2—C23—C24	108.81 (11)
C12—C11—H11	119.8	O1—C23—H23A	109.5
C11—C12—C7	121.26 (15)	O2—C23—H23A	109.5
C11—C12—H12	119.4	C24—C23—H23A	109.5
C7—C12—H12	119.4	C23—C24—C25	111.50 (11)
C18—C13—C14	117.58 (12)	C23—C24—H24A	109.3
C18—C13—C19	123.54 (12)	C25—C24—H24A	109.3
C14—C13—C19	118.71 (12)	C23—C24—H24B	109.3
C15—C14—C13	121.43 (13)	C25—C24—H24B	109.3
C15—C14—H14	119.3	H24A—C24—H24B	108.0
C13—C14—H14	119.3	C24—C25—H25A	109.5
C14—C15—C16	120.50 (13)	C24—C25—H25B	109.5
C14—C15—H15	119.8	H25A—C25—H25B	109.5
C16—C15—H15	119.8	C24—C25—H25C	109.5
C17—C16—C15	118.78 (13)	H25A—C25—H25C	109.5
C17—C16—H16	120.6	H25B—C25—H25C	109.5
			
C6—C1—C2—C3	1.3 (2)	C6—C1—C19—C7	−28.71 (17)
C19—C1—C2—C3	173.68 (12)	C2—C1—C19—C7	159.22 (12)
C1—C2—C3—C4	−0.3 (2)	C6—C1—C19—C13	91.00 (14)
C2—C3—C4—C5	−0.6 (2)	C2—C1—C19—C13	−81.08 (14)
C3—C4—C5—C6	0.6 (2)	C6—C1—C19—C20	−149.52 (12)
C4—C5—C6—C1	0.4 (2)	C2—C1—C19—C20	38.41 (16)
C2—C1—C6—C5	−1.3 (2)	C18—C13—C19—C7	−136.57 (13)
C19—C1—C6—C5	−173.61 (12)	C14—C13—C19—C7	48.34 (16)
C12—C7—C8—C9	−0.2 (2)	C18—C13—C19—C1	101.94 (14)
C19—C7—C8—C9	−174.13 (13)	C14—C13—C19—C1	−73.15 (14)
C7—C8—C9—C10	−0.1 (2)	C18—C13—C19—C20	−17.26 (17)
C8—C9—C10—C11	0.3 (2)	C14—C13—C19—C20	167.65 (12)
C9—C10—C11—C12	−0.2 (2)	C7—C19—C20—C21	−68.12 (14)
C10—C11—C12—C7	−0.2 (2)	C1—C19—C20—C21	56.15 (15)
C8—C7—C12—C11	0.4 (2)	C13—C19—C20—C21	170.60 (11)
C19—C7—C12—C11	174.28 (13)	C7—C19—C20—C22	54.53 (14)
C18—C13—C14—C15	0.9 (2)	C1—C19—C20—C22	178.81 (11)
C19—C13—C14—C15	176.25 (12)	C13—C19—C20—C22	−66.75 (14)
C13—C14—C15—C16	−0.5 (2)	C23—O1—C21—C20	−58.77 (14)
C14—C15—C16—C17	0.3 (2)	C22—C20—C21—O1	54.60 (14)
C15—C16—C17—C18	−0.5 (2)	C19—C20—C21—O1	−179.15 (10)
C14—C13—C18—C17	−1.1 (2)	C23—O2—C22—C20	61.61 (14)
C19—C13—C18—C17	−176.24 (12)	C21—C20—C22—O2	−56.23 (14)
C16—C17—C18—C13	0.9 (2)	C19—C20—C22—O2	176.77 (10)
C8—C7—C19—C1	−39.96 (17)	C21—O1—C23—O2	62.29 (13)
C12—C7—C19—C1	146.35 (12)	C21—O1—C23—C24	−178.24 (11)
C8—C7—C19—C13	−155.31 (12)	C22—O2—C23—O1	−63.62 (13)
C12—C7—C19—C13	31.00 (17)	C22—O2—C23—C24	176.67 (11)
C8—C7—C19—C20	82.92 (15)	O1—C23—C24—C25	176.55 (12)
C12—C7—C19—C20	−90.77 (15)	O2—C23—C24—C25	−63.14 (15)
